# Fluorescence Polarization-Based Bioassays: New Horizons

**DOI:** 10.3390/s20247132

**Published:** 2020-12-12

**Authors:** Olga D. Hendrickson, Nadezhda A. Taranova, Anatoly V. Zherdev, Boris B. Dzantiev, Sergei A. Eremin

**Affiliations:** 1 A.N. Bach Institute of Biochemistry, Research Center of Biotechnology of the Russian Academy of Sciences, 119071 Moscow, Russia; ohendrickson@inbi.ras.ru (O.D.H.); taranovana@gmail.com (N.A.T.); dzantiev@inbi.ras.ru (B.B.D.); saeremin@gmail.com (S.A.E.); 2 Department of Chemical Enzymology, Chemical Faculty, M.V. Lomonosov Moscow State University, 119234 Moscow, Russia

**Keywords:** fluorescence polarization, immunoassay, rotation of molecules, bioreceptors, antibodies, aptamers, nucleic acids, switched on biosensors, portable optical detectors

## Abstract

Fluorescence polarization holds considerable promise for bioanalytical systems because it allows the detection of selective interactions in real time and a choice of fluorophores, the detection of which the biosample matrix does not influence; thus, their choice simplifies and accelerates the preparation of samples. For decades, these possibilities were successfully applied in fluorescence polarization immunoassays based on differences in the polarization of fluorophore emissions excited by plane-polarized light, whether in a free state or as part of an immune complex. However, the results of recent studies demonstrate the efficacy of fluorescence polarization as a detected signal in many bioanalytical methods. This review summarizes and comparatively characterizes these developments. It considers the integration of fluorescence polarization with the use of alternative receptor molecules and various fluorophores; different schemes for the formation of detectable complexes and the amplification of the signals generated by them. New techniques for the detection of metal ions, nucleic acids, and enzymatic reactions based on fluorescence polarization are also considered.

## 1. Introduction

Currently, various analytical systems are being actively developed and widely used for the detection of various substances based on their ability to bind to selective receptor molecules (antibodies, aptamers, lectins, etc.) and to generate a detectable signal induced by this binding [1,2,3,4]. Such a signal can be a change in color, fluorescence, conductivity, or another property, induced by a label included in the detected complexes. In most cases, for effective detection of the label, analysis formats are implemented that include the separation of the detected complexes from unreacted labeled molecules or components of the tested sample that can affect the analytical signal. Attaining this separation requires using various carriers and multi-stage manipulations such as centrifugation, washing, which makes the analysis time consuming and laborious. In this regard, homogeneous non-separating bioanalytical test systems have undoubted advantages. However, distinguishing the bound and unbound labels in the sample medium is not always easy. This problem can be successfully solved by fluorescence immunoanalytical methods based on changes in fluorescence intensity, primarily from the effect of fluorescence resonance energy transfer or fluorescence polarization (FP) [5,6]. The mode based on the FP registration seems more efficient because it depends less on the individual properties of the interacting reagents.

The principle of fluorescence polarization immunoassay (FPIA) has been successfully applied to many analytical problems. Back in 1989, a review of FPIA developments listed 195 references [7]. The FPIA methodology has been successfully implemented in numerous commercial analytical systems. FPIA’s well-studied capabilities and limitations have made it a common method that occupies a strictly defined niche in a number of bioanalytical methods [5,8]. Zhang et al. [5] in a comprehensive review presented its detailed characterization as a means of detecting chemical contaminants in food and environmental analyses.

However, recent developments have indicated that FP can be successfully applied in many analysis formats other than a traditional FPIA. Further development of this topic requires systematization and comparative assessment of recently proposed innovations. Such analysis is the subject of this review, which gives priority to publications of the last five years.

## 2. Physical Bases of Fluorescence Polarization

**Figure 1:**
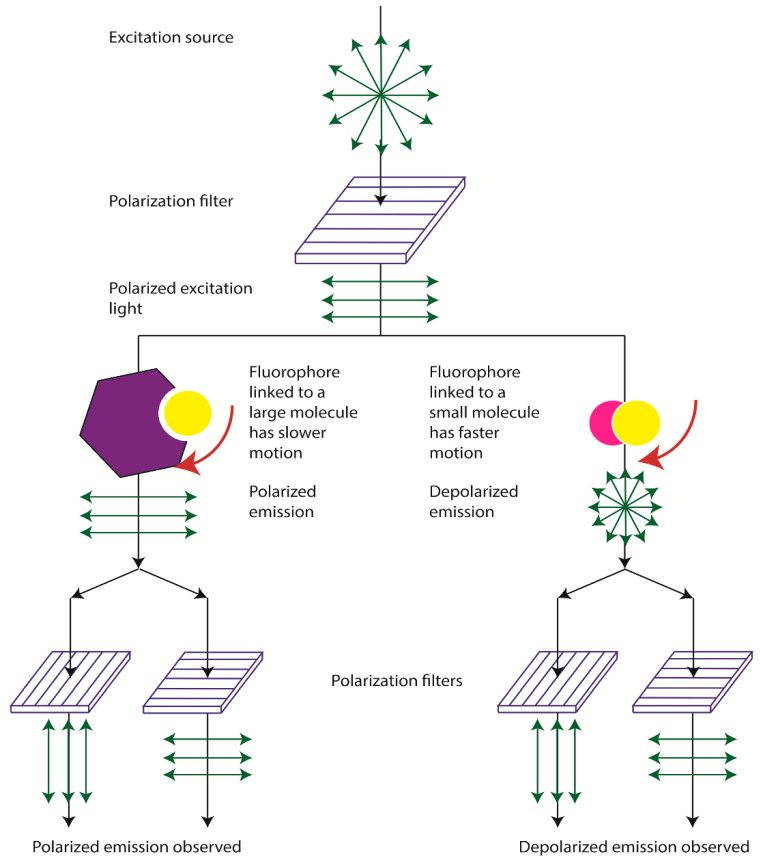
Principle of fluorescence measurement under irradiation with plane-polarized light.

The analytical use of FP is based on irradiating a reaction mixture containing fluorophore-labeled molecules with plane-polarized light and recording the fluorescence that this irradiation induces. In a solution of disordered molecules, polarized light will preferably be absorbed by those whose absorption oscillators are parallel to the plane of polarization. If the excited molecule does not change its orientation in space before emission, the fluorescence emission will also be polarized (Figure 1).

The degree of FP in such a system is described by the Perrin equation (Equation (1)):

1/Р = 1/Рo + [1/Рo − 1/3] × [RT/V] × τ/η(1)

where Р is the registered polarization, Рo is the maximum polarization, T is the absolute temperature, R is the gas constant, η is the viscosity, τ is the average lifetime for of the excited state of fluorophore, and V is the molar volume of the fluorescent substance.

The rate at which the excited molecule changes its orientation in space depends on the rotational relaxation time (φ). This parameter is related to the viscosity of the medium (η), absolute temperature (T), molecular volume (V), and gas constant (R) by Equation (2):

φ = 3η × V/R × T(2)

Therefore, under fixed viscosity and temperature, the FP is directly proportional to the molecular volume. The change in this volume may occur through the binding or dissociation of molecular complexes, through decomposition, or through conformational changes in the molecule. Small molecules in an aqueous medium rotate very quickly and, between absorption and emission, are equally likely to assume any orientation, which leads to a complete depolarization of emission. Large molecules and intermolecular complexes partially retain the same orientation that they had upon absorption of light even during emission [9]. Therefore, their fluorescence is largely polarized. The FP is characterized by the value of P:

Р = (Iv − Ih)/(Iv + Ih)(3)

where Iv is the vertical component of fluorescence (parallel to the excitation beam) and Ih is its horizontal (perpendicular) component.

Another parameter associated with the FP is also used, namely the fluorescence anisotropy (FA), which is determined by the following equation:

FА = (Iv − Ih)/(Iv + 2Ih).(4)

Like any analytical method, FPIA has some drawbacks. First, the sensitivity of the FPIA is lower than that of other immunochemical methods. The FPIA results are affected by the matrix of samples. Special devices are required to register FP and FA.

The probability of polarized light absorption depends on the fluorophore dipole plane’s angle of inclination to the light polarization axis. Photoselection causes the depolarization of 3/5 of the light flux. The angular displacement of the absorption/emission dipoles inside the fluorophore depends on its structure and also leads to light depolarization. Thus, even if the fluorophore molecules are immobile, the FP/FA cannot fulfill Equation (1). For any fluorophore arbitrarily distributed in solution, upon single-photon excitation, the FP varies in the range of 0.333–0.5, and the FA varies from −0.20 to 0.40 [9].

The third parameter that makes a significant contribution to depolarization is the mobility, or, in other words, the rotational diffusion of fluorophore molecules in solution. It is the rotational diffusion of the fluorophore that is sensitive to complexation processes. Because of their high mobility in solution and small molecule size, low molecular weight fluorophores have a high rotational diffusion coefficient. When a low-molecular-weight fluorophore is irradiated with plane-polarized light, the lifetime of the excited state is much longer than the time required for changing the orientation of the molecule in space. Modulation of this parameter is the basis for the development of the fluorescence polarization immunoassay—see Section 3.

## 3. Conventional FPIA, Its Advantages and Drawbacks

**Figure 2:**
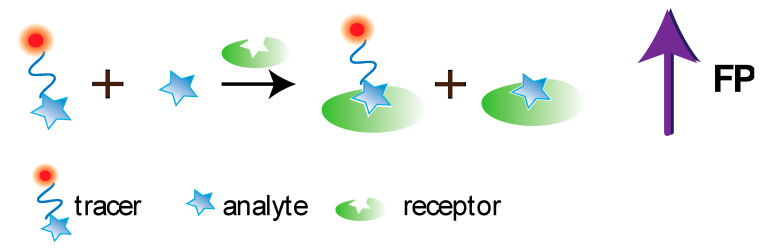
Principle of conventional FPIA.

FPIA was first proposed by Dandliker et al. [10]. Traditional FPIA is based on competitive binding of specific antibodies with the tested analyte and an analyte labeled with a fluorescent label (tracer). The reaction mixture is excited with polarized light, and the fluorescence emission is measured in the direction perpendicular to the excitation beam. Upon excitation with linearly polarized light, the tracer will fluoresce with depolarized light because, by the time of emission, the tracer molecules in the solution are oriented randomly. For the antibody–tracer complex, rotation slows down significantly, providing residual polarization of the emitted light (Figure 2). Therefore, the P value depends on the ratio of bound and free forms of the tracer and is determined by the concentration of the tested analyte.

**Figure 3:**
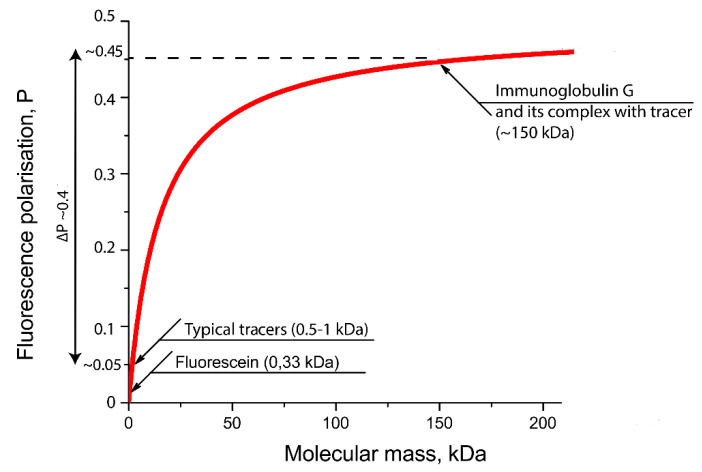
Dependence of the FP of the molecular weight of FITC-labeled preparations calculated on the basis of the Perrin equation (the formula and parameters for drawing are taken from [11]).

The degree of FP change depends on the label, the average lifetime of the molecule in an excited state, the molecular weight of the analyte and the nature of the complex. Therefore, the choice of a fluorescent label is of key importance. It must have a high fluorescence intensity (i.e., high quantum yield and large extinction coefficient), be chemically and photostable under analytical conditions and easily conjugated to the analyte, and not interfere with the ligand–receptor interaction. For the endogenous fluorescent quenchers present in the sample not to interfere with the determination, their excitation and emission wavelengths must differ from the wavelengths of the fluorescent label. In addition, to reduce the effect of light scattering, the label must have a large Stokes shift [5]. When the temperature and viscosity of the solution are constant, the FP will depend only on the size of the fluorescent molecule. In the case of a competitive assay, the higher the receptor concentration, the higher the ligand concentration required to prevent the formation of the labeled ligand–receptor complex, which leads to a deterioration in the assay sensitivity [5]. Accordingly, to achieve maximum sensitivity, it is necessary to use the minimum amount of the receptor, which ensures reliable registration of the FP change during the transition of the labeled ligand into the complex with the receptor. The most commonly used fluorophore is fluorescein isothiocyanate (FITC). The inclusion of FITC-containing derivative of antigen into immune complexes leads to significant changes of FP as can be seen from Figure 3.

FPIA is a homogeneous analytical method that does not require separating the resulting compounds. It allows for determining the analyte concentration within a few minutes.

The method of FPIA is characterized by many advantages inherent in immunochemical methods: for example, versatility for determining low molecular weight substances (for many of which specific antibodies have already been obtained) or the ability to determine either single compounds or a group of compounds depending on the specificity of the antibodies used. Moreover, FPIA has the following unique features:

- It is a simple homogeneous method that requires no washing and separation steps.
- The analysis time is limited only by the pipetting because the kinetic constant of binding of small analyte molecules to antibodies in solution usually varies from 10^7^ to 10^8^ 1/M • s.
- The P value is a relative and dimensionless parameter that smooths fluctuations in instrument measurements and leads to very high reproducibility of results. The variation coefficients usually do not exceed 3–5%.
- Fluorescent labels can be synthesized quite easily and remain stable during storage for many years.
- However, the following features of conventional FPIA should be considered as its drawbacks.
- The assay typically needs in high concentration of reactants for reliable measurements of the FP; due to this FPIA is less sensitive as compared with other immunoanalytical techniques.
- Some compounds of the matrix can absorb excitation and emitted light, as well as exhibit their own fluorescence in the same spectral region as the used fluorophore; as a result, measurement results are distorted.
- The technique may be applied only to detect low molecular weight compounds, as well as it is based on significant difference of molecular weights for fluorophore-labeled antigen and its complex with antibody.

## 4. Modes of FPIA

### 4.1. Single-Reagent FPIA

**Figure 4:**
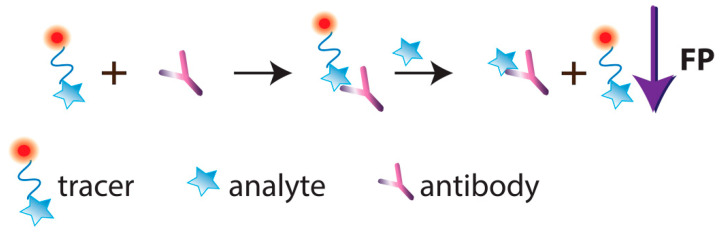
Schematic illustration of single-reagent FPIA.

A specific mode of FPIA is so-called single-reagent FPIA, which uses a tracer–antibody complex. After adding such a reagent to the tested sample, the analyte displaces the tracer from the complex, which is detected as a FP decrease (Figure 4). 

The assay duration and the minimum detectable concentration for the single-reagent FPIA are less than those for the traditional method. The single-reagent method is extremely simplified, the immune complex is significantly more stable during storage, and the calibration curve can be used for quantitative determinations for several days without recalibration [5]. It has been shown that the immune complex can be stored at room temperature for several months. Moreover, the single-reagent FPIA provides a stable calibration curve that can be used over a long period. In this regard, for the determination of an analyte in one sample, there is no need to obtain a new calibration curve, as in other immunochemical methods.

However, the applicability of this approach is limited. Because the single-reagent FPIA is based on the tracer’s displacing from its complex with antibodies, analytical systems with high-affine antibodies cannot be transformed to the single-reagent FPIA. And even in the cases of antibodies having a high kinetic dissociation constant, a displacement of the tracer is a time-consuming step (about 10–30 min), which leads to a loss of the assay rapidity [12]. Therefore, single-reagent FPIA is acceptable, if the content of target analyte in tested sample is in the range of micrograms per milliliter.

Developments of the singe-reagent FPIA include Lippolis et al. on the simultaneous determination of several mycotoxins in wheat [13], Mi et al. on multiple determination of (fluoro)quinolone antibiotics in foodstuffs [14], and Eremin et al. on the high throughput determination of aromatic compounds [15].

### 4.2. FPIA in the Kinetic Mode

Another FPIA format is the analysis in the kinetic mode, when the FP is measured during the first seconds of the interaction and the background signal is absent. Because the kinetics of antigen-antibody interaction in solution is very fast, it can be recorded in the so-called stopped-flow FPIA mode.

A pioneering study in this field was the development of FPIA for 2,4-D and atrazine pesticides in milk and wine without sample preparation [16,17]. The approach used for 2,4-D determination consisted in registering the FP at the initial moment of the immune reaction upon injection of solutions from two syringes, respectively containing a mixture of solutions of the tracer and free analyte and antibodies’ solution. After an injection, the fluorescence intensity was measured at 484 and 520 nm excitation/emission wavelengths.

Sometimes the kinetic mode improves the FPIA sensitivity by an order of magnitude [18]. This effect can be interpreted as the result of measurements at the beginning of the antibody-tracer reaction. Therefore, the interferences from non-specific interactions with matrix compounds are minimized, and FP changes caused by low concentrations of the target analyte can be detected.

Recent studies include, in particular, Reiner et al. [19], who established a kinetic FP-based method for investigating binding kinetics of prominent inverse agonists of histamine. Here, monitoring of association rates of the H3 receptor with its several ligands (ciproxifan, clobenpropit, thioperamide and pitolisant) was carried out. It was demonstrated that the affinities for the ligands estimated in this real-time approach differ from affinities reported in displacement assays. Wolfe et al. [20] proposed a spectroscopic method that requires protein concentrations in the nanomolar range to study the interaction of detergents with membrane proteins. The developed approach is based on steady-state FP spectroscopy, which kinetically resolves detergents’ dissociation from membrane proteins and protein unfolding. A single-fluorophore approach using a microplate reader was developed that could obtain a fast and scalable readout of the protein-detergent interactions at nanomolar protein concentrations.

### 4.3. FPIA in Organic Solvents

In another mode, FPIA uses inverted surfactant micelles (such as Aerosol OT), which is considered promising for reducing the matrix effect. Inverted micelles of surfactants with incorporated proteins are capable of forming optically transparent solutions in non-polar organic solvents. Antibodies in micelles retain their antigen-binding capacity. The main advantage of carrying out FPIA in this way is that the sample can be kept in a non-polar organic solvent, and the immune reaction takes place within micelles in an aqueous solution. Using undiluted extracts of samples in organic solvents enables reduction of the LoD in comparison with the analysis in an aqueous medium. This is especially important for determining compounds poorly soluble in water in such objects as soil and foodstuffs. This approach was extensively used for the detection of pesticides [21,22]. Abd El-Hay and Belal [23] developed a simple high-throughput micelle-enhanced fluorometric technique to determine the presence of antihelminthic niclosamide. To fulfill the assay, the nitro group of niclosamide was reduced to an amino group by Zn/HCl to form a highly fluorescent derivative with excitation at 275 nm and emission at 444 nm. As fluorescence enhancers, carboxymethylcellulose (CMC) and Tween-80 were used. After optimization, the test system allowed for the determination of niclosamide with LoDs of 0.01 and 0.008 μg/mL on using Tween-80 and or CMC, respectively. The approach was tested for the determination of niclosamide in plasma and medicines.

### 4.4. Non-Competitive FPIA

**Figure 5:**
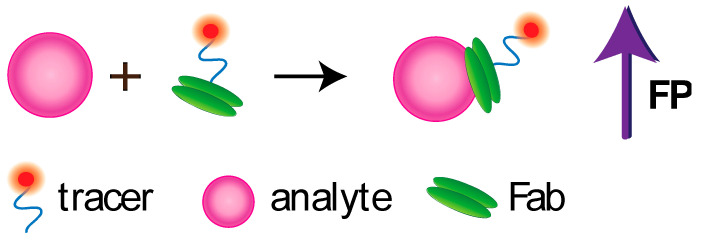
Noncompetitive FPIA using Fab fragment.

Several works are devoted to the development of non-competitive FPIA. Note that non-competitive format for detecting low molecular weight compounds is a great challenge because small molecules cannot significantly increase the volume and the rotation rate of a fluorophore-labelled receptor (Figure 5). 

Fukuyama et al. [24] reported a single-step FPIA of human and rabbit immunoglobulins (IgG) in serum using the variable domain from a camelid VHH antibody. The test system was characterized by a large signal/background ratio in comparison with the previously reported methods using Fab fragments, and the sensitivity was 12-fold. The authors stated that this approach was applicable also to nanometer-sized biological targets, such as viruses and bacteria. Wang and Si proposed a direct non-competitive FP aptasensor that did not require florescent analogues for determining adenosine [25]. Adenosine, working as a molecular linker, reassembled the two pieces of single-stranded DNA into the intact aptamer tertiary structure, and then induced the increase of the FP signal. The LoD of adenosine was 26 nM; a wide dynamic range of over five orders of magnitude (50 nM to 1.0 mM) was achieved. Zhao et al. [26] used aptamer-based non-competitive FA analysis of OTA. A TMR-labeled aptamer demonstrated a FA reduction response upon binding with OTA; therefore, no FA signal enhancers were required. The developed test system showed a LoD of 3 nM and a dynamic range of 3–3000 nM and allowed for the measurement of OTA in urine and red wine. Geng et al. described a FA strategy that did not need a DNA aptamer or OTA labeling with fluorophores [27]. The fluorescence properties of OTA were considered in developing the test system. The interaction of OTA with the aptamer led to the deceleration of OTA rotation, thus enhancing the FA of OTA. Nishiyama et al. [28] reported the development of a non-competitive FPIA for C-reactive protein (CRP) using HiLyte Fluor™647 as a far-red emitting fluorescent dye to label CRP-specific antibody Fab fragment. The assay of human serum can be implemented in 10 min without sample preparation and has the cut-off value of 10 μg/mL.

## 5. FP-Based Assays with Alternate Recognizing Elements

### 5.1. Aptamer-Based FPA

Recently, FP techniques using aptamers, molecularly imprinted polymers (MIPs) and proteins as alternative receptor systems have been developed [8,27,29,30]. Among the demanded recognition elements are aptamers, single-stranded oligomers of nucleic acids capable of selectively binding a variety of targets from low molecular weight organic substances to high molecular weight molecules, such as proteins. The production of aptamers of a given specificity in large quantities became possible through the creation of the Systematic Evolution of Ligands by Exponential Enrichment (SELEX) technology, a method of in vitro directed selection of sequences with specified properties from a combinatorial oligonucleotide library. Regarding the nucleic chemistry of aptamers, there is no preference between DNA or RNA to produce aptamers. Comparing the dissociation constants for RNA and DNA aptamers indicates that their affinities are comparable [31,32].

Aptamers have several advantages over antibodies. The production of new aptamers occurs in completely in vitro conditions from a combinatorial library [33]. The researcher can control the process of selection of the final aptamers by changing the interaction conditions at different stages [34]. Moreover, the affinity of the aptamers is not inferior to that of antibodies [31]. Any aptamer with known sequence can be easily synthesized, which significantly reduces its cost. The aptamer structure’s simplicity enables its targeted modification with the necessary functional groups [34]. Unlike antibodies, aptamers can completely renature without loss of ligand-binding properties after exposure in such denaturing conditions as high temperature or ionic strength [35].

The most critical drawback of aptamers is the limitation associated with the structure of nucleic acids. The affinity and specificity of the ligand–receptor interaction depend both on the variety of receptor’s conformations and on its functional groups [36]. Nucleic acids have less diverse functional groups than proteins. In particular, the absence of hydrophobic groups negatively affects the specific interactions with ligands rich in hydrophobic regions [37]. To increase the diversity of functional groups, additional pre-SELEX chemical modifications are implemented [37]. Besides, the usage of aptamers for the analysis in biological samples, e.g., serum, is limited by their rapid degradation by nucleases [38]. Since oligonucleotides’ decay in blood can occur within several minutes, their application for non-treated samples is questionable and needed additional solutions. Another drawback of aptamers is that their affinity depends on the pH and composition of the reaction medium, therefore, aptamer can loss its affinity in complex matrixes [39].

**Figure 6:**
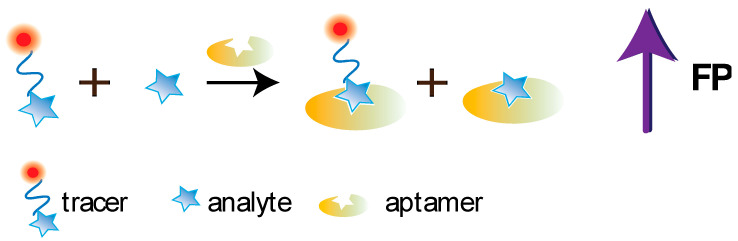
Principle of aptamer-based FPA.

At present, a number of studies have been devoted to the development of FPA using aptamers; the main achievements in this area are summarized in several reviews [8,27,40]. The principle of the competitive FPA using aptamers is demonstrated at the Figure 6.

Table 1 lists the studies considered in this review. No investigations have been undertaken on the determination of low molecular weight compounds by the aptamer-based FPA in complex matrices as in traditional FPIA. However, different alternative schemes of aptamer-based FPA have proliferated [41]. The absence of competitive FPA is probably because the molecular weight of aptamers is significantly smaller than that of antibodies: most aptamers are no more than 60–80 nucleotides long [31]. The molecular weight of an aptamer with a 60- nucleotide chain length is less than 20 kDa, whereas the weight of an antibody (IgG) is about 150 kDa. Therefore, the formation of an aptamer–fluorophore complex will be accompanied by a smaller change in fluorophore mobility and a smaller FP change than the formation of a complex of the same fluorophore with an antibody [42].

Test systems where either the aptamer itself or a complementary short-labeled oligonucleotide acts as a labeled compound can be seen as alternative formats. Thus, Liu and Zhao [43] developed a simple and rapid analysis of bisphenol A (BPA) with the FA measurement using a tetramethyl rhodamine (TMR) and labeled short DNA aptamer to BPA. The analysis is based on a conformational change of the labeled aptamer caused by the binding with BPA and on a change in the interaction between guanine bases and TMR, which leads to a change in FA. The LoD of BPA in tap water was 0.5 μM.

A TMR-labeled aptamer probe was also used in the study by Liu et al. [44] devoted to the analysis of cocaine based on FA registration. The cocaine interaction with an aptamer leads to a change in the structure of the TMR-labeled aptamer, which leads to changes in the binding between the labeled TMR and adjacent G bases in the aptamer sequence. Therefore, an increase in the cocaine amount results in the FA changes of TMR. Optimizing the assay conditions enabled the determination of cocaine with up to 5 μM LoD in urine and diluted serum samples. Chevelon et al. [45] used a labeled oligonucleotide complementary to the aptamer to detect D- and L-arginine vasopressin.

Based on the FP theory (see Section 2), FP changes, which occur during complexation, depend on both fluorophore and receptor sizes. The larger the difference in their sizes, the more pronounced will be the FP change detected upon fluorophore transition from free to bound state. The ligand size is unchangeable and depends on the selected target, whereas the size of the receptor or the ligand-receptor complex may vary. This idea was reflected in a number of publications devoted to aptamer-based FPA. An additional change in the molecular weight of the labeled component was achieved by various manipulations depending on the ligand’s presence or absence (for example, analyte-dependent cleavage of the labeled aptamer by DNAses). Thus, Kang et al. proposed a rather complex scheme for FP-based detection of adenosine [46]. The aptamer labeled with a fluorescent dye and biotin at the 5′-end group interacted with adenosine, after which DNAse was added. Binding to adenosine shielded the aptamer from DNAse cleavage, while all unbound sequences were cleaved, releasing a fluorescent label. After this, streptavidin (SA) was added, which bound to the complex of aptamer with adenosine. It was demonstrated that the use of SA allows a tenfold decrease in the LoD to as low as 0.5 μM. In the study of Li et al. [47], a strategy of molecular mass amplifying was developed for miRNA determination in a biological matrix. The probe loses its activity because of a small blocking strand having partial complementarity to both aptamer and the miRNA sequences. Therefore, only activation of the probe by the target miRNA will promote binding of the aptamer domain to the protein. The developed approach for miRNA detection was characterized by high sensitivity (LoD was 3.4 pM), selectivity, and a short analysis time. The change in mass can also be achieved through ligand-dependent cleavage of the label by exonuclease from the labeled oligonucleotide immobilized on nanoparticles and partially complementary to the used aptamer. Huang et al. [48] used this technique for the detection of ochratoxin A (OTA) with a femtomolar sensitivity.

As an alternative to catalytic cleavage, other approaches have been proposed for increasing the receptor mass. Samokhvalov et al. [11] developed a sensitive FP analysis of OTA where an aptamer as the receptor was included in complexes with protein modules to enhance its size. Fluorophore-labelled and free OTA competitively interacted with aptamer-based receptors. The binding of labelled OTA with aptamer–anchors complexes led to the increase of a FP in comparison with that resulting from binding with a free aptamer. Accordingly, a 40-fold gain in the assay sensitivity was achieved. The OTA LoD in red wine was 1.1 µg/kg (2.8 nM). Cui et al. [42] proposed a direct FPA of adenosine and cocaine using a fluorescently labeled chimeric aptamer consisting of cocaine/adenosine and thrombin recognition sites. Chimeric aptamers were designed in such a way that upon adenosine or cocaine binding, a thrombin-recognition site was released. This led to the binding of the aptamer to thrombin (MW = 40 kDa), which was accompanied by a significant change in the FP of the label. Huang et al. proposed a scheme for adenosine detection using a fluorescein-labeled chimeric aptamer [49]. Conformational changes of the labeled chimeric aptamer in the presence of adenosine underlie the principle of the assay. Accordingly, it could specifically hybridize with the complementary sequence immobilized on silica nanoparticles, thereby causing a significant change in the FP of the label.

### 5.2. FPA Based on Molecularly Imprinted Polymers

**Figure 7:**
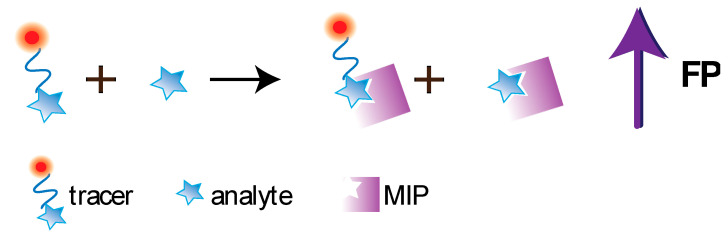
Principle of the competitive FPA using MIP.

Molecularly imprinted polymers (MIPs) are increasingly being used in analytical chemistry as synthetic recognition elements [29,30]. Molecular imprinting represents a technology aimed at introducing recognition properties into synthetic polymer through synthesis of a network with molecularly imprints as specific molecular recognition sites. MIP-based analytical methods are often equal or superior in sensitivity and specificity to immunoassays [29,30]. Among MIPs’ advantages are the methodological simplicity of obtaining receptors for low molecular compounds, rapid MIP production, highly reproducible synthesis, possibility of conducting MIP-based analysis in the organic media, imprinting of highly toxic compounds, and stability in harsh conditions. However, obtaining MIPs for biomolecules having high molecular weight (and, accordingly, the development of their MIP-based assay) is still a methodological problem. Further, the polymer’s availability of molecular recognition sites, which are located within the macroporous structure, is limited for high molecular weight compounds. In addition, the analyte-imprinted polymer reaction often occurs more efficiently in organic solvents. Besides, MIPs’ binding sites are characterized by high heterogeneity in both affinity and specificity, which can be also be considered as disadvantages. The principle of the competitive FPA using MIP is demonstrated at the Figure 7.

Few MIP-based FPAs can be found in the literature. It may be explained by the difference between the structure of the fluorescently labeled analyte and that of the template molecule can be an obstacle to assay implementation. Studies on FPA using MIP technology included the development of 2,4-D and (*S*)-propranolol assays [50,51]. The IC_50_ value for 2,4-D was 10 mM. The FPA of propranolol allowed for the stereospecific determination of (S)-propranolol because the MIPs used were obtained for this stereoisomer. Ton et al. [52] developed FPA based on water-compatible MIP nanoparticles for the detection of enrofloxacin (ENR) and structurally similar antibiotics from a fluoroquinolone group. Because ENR is a UV-excited fluorescent compound, its direct quantitative detection could be performed without separation of free antibiotic from the MIP-bound one. This rapid and robust FPA was applied for detecting ENR in milk and tap water without a preconcentration step. The LoD for ENR was 0.1 nM. Murase et al. [53] synthesized core-shell MIP nanoparticles for FP-based determining cortisol. A competitive FPA was carried out using free and dansyl-labeled cortisol. The achieved LoD was 80 nM. Xiao et al. [54] developed a MIP-based FPA for micromolar determination of diclofenac in water based on a cyclodextrin polymer having an inherent affinity for the target analyte. The detected pharmaceutical displaced a fluorescein sodium salt as a dye adsorbed in the cyclodextrin-based polymer, resulting in the growth of the FP of the released dye. The test system allowed for the detection of diclofenac in wastewater with a LoD of 1 M.

### 5.3. FPA Based on Other Recognizing Elements

If the immune recognition underlying FPIA is not principled as a ligand–receptor interaction, and the task of the study is to assess the functional activity of a wide class of compounds, other receptors could be used, such as enzymes or other proteins. Currently, methods similar to FPIA for determining many biologically active compounds have been developed. For example, such a technique was proposed for detecting phthalate esters (PAEs) in spirits [55]. A fluorescent-labeled fatty acid (C4-BODiPY-C9) and a recombinant peroxisome proliferator-activated receptor α ligand binding domain were used as a receptor and a fluorescent tracer, respectively. LoDs of 10 PAEs were below 11.2 μM. A series of studies was devoted to the detection of BPA and its analogues as environmental endocrine-disrupting compounds [56,57,58]. A competitive FPA based on glucocorticoid receptor as a receptor and a dexamethasone fluorescein (Dex-FL) as a dye was developed for detecting BPA analogs in soybean oil [57]. The established LoD of BPA was 1.0 µM with a working range of 7.6–30 µM. The result of FPA showed a good correlation with that of HPLC. In the next study, a FPA was developed to simultaneously determine BPA and its derivatives in soil samples [56]. As a recognition element, a recombinant peroxisome proliferator-activated receptor alpha ligand binding domain (mPPAR alpha-LBD*) was applied. The receptor was characterized by broad specificity toward bisphenols, allowing for the detection of all tested compounds with LoDs of 0.21–1.03 µg/g.

A protein receptor-based FPA seems promising also for studying ligand–receptor interactions important for drug and vaccine development. Allikalt et al. [59] developed a fluorescent FA assay to assess interaction of BODiPY-FL-SKF83566 fluorescent ligand with dopamine D-1 receptors, which are substantial regulators in the brain and therefore important targets in drug discovery. The approach enabled the assessment of kinetic properties and the affinity of an unlabeled ligand based on the competition binding analysis and had a good correlation with radioligand-based methods. Yao et al. [60] synthesized a FP probe based on cyclic peptide specific to influenza hemagglutinin (HA), a virus surface glycoprotein used for development of novel vaccines, therapy, and production of antibodies with broadly neutralizing properties. The developed assay allowed high-throughput screening to find molecules having high affinity to HA of influenza A. The authors hoped that their investigation could contribute to the discovery and design of new powerful antiviral agents. The use of receptor-specific peptides as competing agents provides possibility to overcome limitation to molecular weight of analytes. The similar approach could be applied for antibody-based assays (see Section 12).

Several studies describing the use of enzymes as a recognition element for FP test systems have been reported recently. Thus, Wang et al. [61] used dihydropteroate synthase as a recognition element for FP multidetection of sulphonamides. The developed assay was characterized by simplicity and rapidity and allowed for a class-specific detection of at least 29 sulphonamides with high sensitivity (IC50 < 100 ng/mL) within 20 min. Alfonso et al. [62] used Na,K-ATPase as a receptor having a high affinity to fycotoxin palytoxin in the development of its FP assay. The fluorescent probe was obtained by coupling Na,K-ATPase with carboxyfluorescein. The LoD of palytoxin was 2 nM. Using Na,K-ATPase as a recognition element excludes the need for immunizing animals with highly toxic compounds to obtain natural antibodies. The authors indicated that the detection method was easier, faster, and more reliable than the other described approaches.

## 6. Fluorescein and Alternative Fluorophores

Fluorescein and its derivatives are still the most frequently used fluorophores in FPIA [5]. Although the Stokes shift of fluorescein is small (23 nm) and, therefore, the fluorescein label is very sensitive to light scattering and background intensity, fluorescein dyes occupy an absolutely dominant position in the detection of various compounds using FPIA. Several reviews summarize the use of fluorescein for detecting various analytes [5,8,63]. The choice of fluorescein dyes can be explained by their excellent spectral properties (fluorescence lifetime is 4.05 ns, quantum yield is 92%), unified filters for absorption/fluorescence wavelengths on all FP analyzers, chemical stability, and the possibility of conjugation with various reactive groups (sulfhydryl, carboxyl and amino groups). Carboxyfluorescein (FAM), FITC, glycylaminofluorescein, fluoresceinamine, 4′-(aminomethyl) fluorescein, 4,6-dichlorotriazinyl aminofluorescein, and aminoacetacetamido fluoresceinamine are the used fluorescein dyes. Fluoresceins are characterized by a large change in the polarization of the emitted light and therefore are especially suitable for the FPIA. It is also necessary to note an insignificant photolability under normal conditions, a low temperature coefficient of fluorescence (the need for temperature control is excluded), and a high fluorescence yield in the bound state (30–60%). When irradiated with blue light (at a wavelength of 492 nm), the fluorescein molecule goes into an excited state, the lifetime in which is 4 nse and emits green light with a maximum wavelength of 517 nm. If a fluorescein molecule is irradiated with plane-polarized light, it will also emit polarized light. The degree of FP will depend on the angle through which the fluorescein molecule rotates during the time between excitation and emission. The rotation time of the molecule through an angle of approximately 68.5° is defined as the rotational relaxation time, which is 1 ns for a small molecule of fluorescein and 100 ns for large molecules such as immunoglobulin. Because the molecules are randomly oriented in the solution, the resulting FP will be approximately equal to zero, and for an antibody–antigen immune complex labeled with fluorescein, FP will be large.

In addition to fluorescein dyes, other labels are used in modern FPIA: rhodamine [45,64,65], BODiPY-FL [4,55], Alexa Fluor [66,67,68], Oregon Green [68], and others, most of which have excitation/emission wavelengths similar to fluorescein. The development of new fluorochromes has a large potential to improve the analytical parameters of the FPIA. However, novel fluorescent dyes should be adopted to the current practice (e.g., dyes must be available, labeling techniques and analysis protocols must be established, and the application of a new dye should lead to sensitivity enhancement). Whether these new labels have advantages over fluorescein is currently not completely clear, but fluorescein tracers are certainly cheaper and easier to synthesize.

Because of its photophysical properties, rhodamine A can serve as a worthy alternative to fluorescein. As a rule, the rhodamine derivative exhibits a color change to pink and emits intense fluorescence in acidic media from activation of the carbonyl group in the spirolactone or spirolactam fragment. Li et al. [64] reported an aptamer FA assay of OTA with the use of lissamine rhodamine B labeled OTA. The analysis is based on a competitive binding between free OTA and its fluorescent derivative with the SA-conjugated aptamer. The LoD of 10 nM was achieved. The developed method allowed detection of OTA in spiked red wine samples, showing the assay’s applicability in complex matrices. Sun et al. developed a direct FA assay for detecting a mycotoxin aflatoxin B1 (AFB1) based on its binding with an aptamer having a single TMR fluorophore on a specific site [65]. Using the TMR-labeled aptamer probe allowed for the achievement of AFB1 LoD of 2 nM and confirmed a high potential in complex sample analysis. Liu et al. [44] described an analysis for cocaine based on aptamer labeled on a specific position with TMR. The assay principle lies in that the interaction of cocaine and aptamer results in structural changes of the TMR-labeled aptamer and, correspondingly, changes in interactions between TMR-labeled and certain G bases in the aptamer sequence. Therefore, a growth of cocaine concentration in the tested sample results in shift of TMR FA. The developed assay enabled cocaine content to be controlled in spiked serum and urine samples; the LoD of cocaine was 5 µM.

The use of the BODiPY-FL dye is described by Guan et al. [55], where BODiPY-FL-NAN-190 was applied to control the ligand binding to 5-HT1A receptors expressed in baculovirus particles followed by an increase in FA. Zhang et al. [69] developed the FPIA for detection of phthalate esters in Chinese spirits, a fluorescent-labeled fatty acid (C4-BODiPY-C9) was employed as a probe. For 10 target analytes, the LoDs in spirits were below 11.2 μM. Tereshchenkov et al. synthesized derivatives of macrolide antibiotics with fluorescein, BODiPY-FL, rhodamine, Alexa Fluor 488, and nitrobenzoxadiazole (NBD) dyes [70]. To assess the binding of these complexes with E. coli ribosomes, FP and the dissociation constants for ribosome with antibiotics were estimated. BODiPY-FL and NBD derivatives were shown to be applicable for screening the binding of bacterial ribosomes with antimicrobials. In Wang et al.’s study [66], Alexa Fluor 488 has attracted much attention for its fluorescence properties, including less photobleaching and pH independence. A nanoscale probe for the rapid and sensitive assay of nucleic acids was developed, constructed from a partially complementary double-stranded DNA (dsDNA) used as a link between Alexa Fluor 488 and gold nanoparticles (AuNPs), leading to the surface energy transfer. Competitive displacement of the probe by the target analyte reduced Alexa488′s mass, and fluorescence recovery reduced the FP, enabling sensitive estimation of the target concentration and thus achieving a pM level detection of single-stranded nucleic acids. Choi et al. [67] used amplified FP assay to measure the affinity of human angiogenin and a single-stranded DNA aptamer. SA interacted with a biotinylated single-stranded DNA aptamer followed by the binding Alexa Fluor 488 labelled human angiogenin. The amplification of FP signal using the biotin-SA module enabled achievement of a LoD of 6.3 nM. Jurewicz et al. [71] used Alexa Fluor 488 dye in a FP approach for estimating a peptide binding constant between an MHC-I β2m subunit and a fluorescently labeled peptide.

Laursen et al. [72] proposed for FPIA a new class of dyes—triangulenium dyes—with a combination of the red emission and a long fluorescence lifetime. They described a preparation and purification of stable triangulenium bioconjugates and discussed aspects of their application of FP methods. Ohashi et al. [73] reported a FPIA based on a Quenchbody (Q-body), single-chain antibody variable fragment labeled with fluorescent dye. Upon binding to the antigen, the fluorescence intensity of the Q-body increased. A Rhodamine 6G-like fluorescent dye, ATTO 520 was used to label anti-BPA Q-bodies. After the antigen was added to the ATTO 520 labeled Q-body, the antigen–antibody reaction resulted to the dye release. Accordingly, more active Brownian motion of the dye caused lower FA.

Interference from scattered light and endogenous fluorophores in samples can lead to an increase in background fluorescence and impair the analytical performance of FPIA. To minimize this phenomenon, use of fluorophores with a longer excitation wavelength than fluorescein, such as complexes of transition metals (e.g., ruthenium and rhenium), has been proposed [63,74,75]. They fluoresce in the 630–700 nm range and have a large Stokes shift—up to 250 nm. The use of these fluorophores allows one not only to reduce the background signal but also to determine high-molecular compounds. Okada and Minoura [74] proposed a fluorescent ruthenium metalloglycocluster ([Ru(bpy-2Gal)(3)]) as a FP probe to study binding between carbohydrates and lectins. Changes in the FP of metalloglycoclusters were detected after the addition of lectins. Thus, after the addition of peanut agglutinin, the FP value of increased. The proposed approach allowed for the measurement of dissociation constants between the metalloglycoclusters and lectins. Sanchez-Martinez et al. describes the determination of gliadins in gluten-free food products at concentrations up to 0.09 μg/mL [75]. A ruthenium(II) chelate as a long-lifetime fluorescent label has been used in FPIA of macromolecules gliadins. During the assay, the target analyte displaces the gliadin-Ru(II) chelate tracer from the complex with an antibody, followed by a decrease in the FP proportional to the gliadin concentration. Lo et al. [76] described rhenium(l) polypyridine maleimide complexes as thiol-specific luminescent labels for bovine and human serum albumins, a glutathione, and thiolated oligonucleotide. These probes demonstrate highly polarized emission, long lifetimes, and high quantum yields, which make them prospects for FPIA of high molecular weight analytes.

Recently, significant progress in FPIA was achieved by introducing nanoparticles as enhancers. Chen et al. [8] described nanomaterial-based FPIAs. Among the fluorescent nanomaterials widely used in biosensorics are quantum dots (QDs). Comparison with organic dyes demonstrates such advantages of QDs as excellent photostability, high quantum yield, and narrow fluorescence spectra with specified maxima. In recent years, QDs have been used in FPIA to detect tumor markers [77,78], cysteine and mercury ions [25], antithrombin [79], and adenosine triphosphate (ATP) [80], among others. In addition to QDs, copper nanoclusters have been used in FPIA to detect the Citrus Tristeza virus [81], AuNPs have been applied as tracers for the sensitive detection of silver ions [23], silica nanoparticles—for thrombin detection [82], and carbon nanoparticles combined with aptamer—for real-time FA detection of apyrase [83], as well as in near-infrared fluorescence dye to detect ciprofloxacin [84].

## 7. FPIA for the Determination of Metal Ions

Another promising field of FPIA is the determination of metal ions. Many techniques are known for quantitatively determining metal ions, but they are quite expensive and require special equipment. In addition, determining metals in biological samples, including those from the environment, requires a large volume of samples and a special pretreatment. FPIA can be proposed as an alternative method. Several studies were devoted to the development of FPIA of silver ions. As is known, small molecules, including metal ions, cannot produce substantial FP changes. Zhang and Wang [85] developed a technique for the detection of Ag^+^ based on a FP reduction. To fulfill this approach, guanine-rich oligonucleotides were labeled by a TMR fluorophore. Formation of the G-Ag^+^-G base pair resulted in the change from the unfolded to a hairpin-like folded structure. This diminished the interaction between guanine and TMR by photoinduced electron transfer and, accordingly, the reduction in FA response. The developed test system enabled measuring of Ag^+^ with a LoD of 0.5 nM and a dynamic range of 2–100 nM.

In Wang et al. [86,87], Ag^+^ ions in aqueous solutions were detected by a FP sensor using AuNPs functionalized by SH–DNA. The assay was based on specific interaction of Ag^+^ with a cytosine–cytosine (C–C) mismatch in DNA duplexes followed by a formation of stable metal-mediated cytosine–Ag^+^–cytosine (C–Ag^+^–C) base pairs and, accordingly, changes in the molecular volume and FP. The developed test system enables determination of Ag^+^ within 6 min at nanomolar levels.

Qi et al. [88] developed a FP-based method for sensitive detection of Ag^+^ based on MnO_2_ nanosheet-assisted and ligand–DNA interaction. The addition of Ag^+^ to the initially formed proflavine-DNA complex resulted in the proflavine release followed by a weak FP change. The subsequent addition of MnO_2_ nanosheets enhanced the FP changes, enabling the quantitative measurement of Ag^+^. The achieved LoD was 9.1 nM with a 30–240 nM linearity. This biosensor could be recommended for environmental water analysis. Jiang et al. [89] developed a highly sensitive and specific FP aptasensor based on signal enhancement by silver nanoparticles (AgNPs) and aimed at mercury ion detection. One of the two T-rich aptamers contained in the aptasensor was labeled with CdTe-CdS QDs; the other was modified with AgNPs as amplifier. Binding of the recognition element with Hg^2+^ as a target analyte led to an apparent FP change. The LoD for Hg^2+^ was 6.6 nM with an analytical range from 10 nM to 0.4 μM.

## 8. FPA of Nucleic Acids

DNA analysis plays a huge role in diagnosing infectious diseases, identifying people in forensic and paternity testing, typing tissues for histocompatibility. In addition, only DNA detection methods allow obtaining unambiguous results in large-scale genetic studies that identify alleles of hereditary diseases [90,91]. The genotyping method is widely used in various formats of highly sensitive and specific analysis [92].

Phenomenon of FP has been used for protein–DNA and protein–protein interaction research, DNA detection by strand displacement amplification, and genotyping by hybridization [93,94]. Until the FP degree stabilizes, the true value increases linearly to 10 kDa in molecular weight. Since the fluorophore-labeled nucleotide has a molecular weight of ~1 kDa, and the fluorescent 25–30 mer—~10 kDa, FP is ideal for fixing the primer extension reaction. Chen et al. [95] proposed using the effect of inhibition of DNA and RNA fluorescence of a cationic dye in the red region of Nile blue (NB). The assumption that dye served as an intercalator to the stack base pairs of DNA or RNA was confirm with different studies such as thermal denaturation, absorption and emission spectra. The linear range for determination of DNA was 3.0 ng/mL–2.0 μg/mL and RNA was 27 ng/mL–10 μg/mL. The LoDs were 3.0 ng/mL and 27 ng/mL respectively. Despite the widespread use of ddNTPs labeled with dye in sequencing reactions [96] and the full-scale study of genotyping methods using the protocol extension of the primer [97], the first use of FP for recording elongation primer was presented only by Chen et al. [95]. These experiments demonstrated the simplicity, high sensitivity, and specificity of FP as a method for recording primer extension by one base pair in a homogeneous reaction. The FP under constant temperature and viscosity of the solvent largely depends on the molecular weight of the dye. Fixation of the FP fluorescent dye allows significant changes in molecular weight of the molecule to be recorded without separation or purification [90]. The results show that both FP and fluorescence are reliable and effective parameters.

Zhu et al. [98] described a new PFA method based on aptamers and FP enhancing by ssDNA binding protein (SSB). The molecules of fluorescein and Texas Red were entered into various parts of the aptamer, which made it possible to obtain 10 fluorescent indicators. The reaction of the aptamer with SSB led to a sharp increase in FP without the target analyte because of the different mobility of the initial SSB molecules and the final SSB-dye complexes. When the target analyte reacted with aptamers, a coiled tertiary structure of the complex was formed, which led to the release of labeled nucleic acids from the protein and a strong decrease in FP. Gaus et al. [99] described the interaction of nucleic acids witch was modified with plasma proteins. They used FP to measure the binding constant of 25 of the most numerous human plasma proteins to phosphorothioate modified antisense oligonucleotides. The study revealed that antisense oligonucleotides, for the most part, actively interact with albumin and glycoprotein, a significant percentage of which is represented by histidines.

Wang et al. [66] demonstrated highly sensitive assay for nucleic acid detection by competitive binding with FP. Partially complementary dsDNA was used as a binder for AuNPs and dye Alexa Fluor 488. This interaction leads to the transfer of surface energy from the dye molecules toward AuNPs. At the same time, a sharp decrease in the actual dye concentration, which reflects the repression of the FP, with a simultaneous increase in the mass or volume of the complex, leads to an increase in the relaxation time of Alexa488 rotation, and, consequently, to increased FP. When there is a competitive substitution between the label and the target nucleic acid, there is a decrease in the mass or volume of the complex with Alexa488, and therefore the FP decreases. This effect can be used to determine nucleic acids at concentrations not exceeding the pM level. Despite the complexity of the flowing reactions, the analysis time does not exceed 30 min.

Nikiforov and Jeong [92] describe an apparatus and method for detecting FP during a nucleic acid reaction such as polymerase chain reaction (PCR) amplification or isothermal amplification. FP can be detected simultaneously in several samples. In addition, several fluorophores can be used to detect different sequences in a sample during the same reaction. Oleksy et al. [100] studied methods for determining water-soluble ligands with a large molecular weight. Ligand patterns compete with fluorescently labeled peptides for the binding. The presence of the ligand and the binding of a partner to it leave the labeled peptides free to manifest the fluorescence depolarization. Wu et al. [101] used FP to study interactions and detect target molecules. In this work, the FP was effectively used to demonstrate the genotyping of the amplification-resistant mutation system. In this group of reactions, allele-specific products of the PCR were recorded by hybridization of amplicon-specific DNA sequences. Despite the reliability and sensitivity of hybridization reactions, implementing this method requires extensive experience in developing target labels and protocols for genotyping applications. In the case of the primer extension reaction and its registration by the FP method, the reaction conditions are universal and require almost no optimization.

## 9. FP in the Monitoring of Catalytic Processes

**Figure 8:**
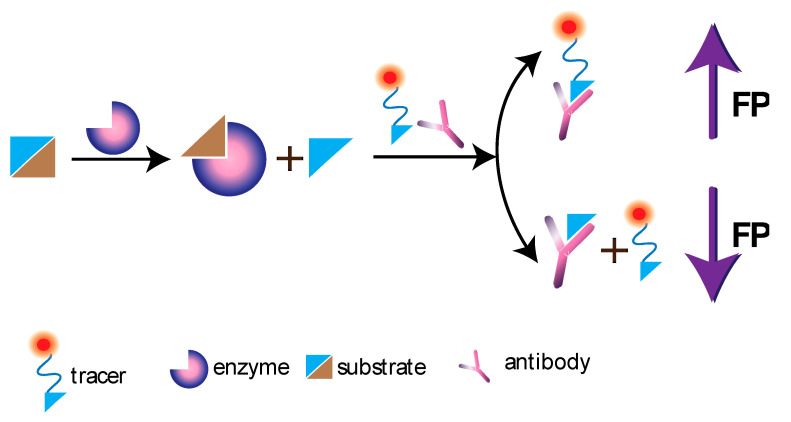
Principle of the using FP to determine enzymatic activity.

Competitive FP assay, which is based on the interaction between nonmodified analytes and fluorescently labeled analogs (labels or indicators) for binding to specific antibodies, is widely used to test enzyme inhibitors as potential new drugs for anticancer and antibacterial therapy. The FP allows to study selective inhibition of enzymatic activity (Figure 8). One of the first works on using FP to determine enzymatic activity was Miura’s 1985 publication [102] about recording the activity of human lysozyme in urine. The assay represented a reaction between an enzyme and a substrate labeled with fluorescein, which caused a decrease in FP. This interaction is hyperbolic in the range of lysozyme concentrations from 0.01 mg/L to 10.0 mg/L.

Fiene et al. [103] presented FP analysis of hydrolase activity. Hydrolases of the ectonucleoside triphosphate diphosphohydrolase family, among which NTPDase1, -2, -3, and -8 should be singled out separately, play an important role in the transmission of purinergic signals, through which the levels of extracellular nucleotides are regulated. The authors have developed FPIA of NTPDase, which is based on the use of ATP or ADP as substrates. The dephosphorylation to ADP is catalyzed by NTPDase1, followed by dephosphorylation to adenosine monophosphate (AMP) as the final product. NTPDase3 and -8 produce AMP and ADP, whereas NTPDase2 leads to a greater extent to the formation of ADP. The FP reaction of detecting the corresponding product, AMP or ADP, is due to the replacement of the corresponding fluorescent indicator nucleotide from the specific antibody. This reaction results in FP changes with LoDs of 20 mM ATP or 10 mM ADP, which is below the Michaelis constant (KM) values of NTPDases.

Kleman-Leyer et al. [104] proposed a method for determining the kinase activity via direct immunodetection of nucleotides using the Transcreener^®^ platform (BellBrook Labs, Madison, Wi, USA). This paper describes the creation of antibodies with selectivity for ADP over ATP over 100-fold, which allows reliable determination of initial rates at ATP concentrations in the range from 0.1 μM to 1000 μM. A method for calculating the KM of ATP kinase using this FP immunoassay is also presented. The proposed method is fundamental in the screening of inhibitors and selectivity agents for any enzyme that produces ADP. Beebe et al. [105] studied and identified patterns between the kinetic functions of phosphorylation of different EGFR substrates of wild-type and oncogenic mutants in vitro. Studies have shown that the studied indicators differ between the EGFR species. The nature of the differences lies in the substrate. The experiments were carried out using FPIA, which efficiently reveals the distinctive nuances of phosphorylation of both peptide and natural substrates. Results are consistent with conventional (γ-32P) ATP/filtration analysis.

Su et al. [106] presented a fast and sensitive assay for methyltransferase using the competitive FP assay. For the same purpose, Graves et al. [107] developed a high-throughput competitive FPIA based on determining the product of all methyltransferase reactions—S-adenosylhomocysteine (AdoHcy)—using S-adenosylmethionine (AdoMet). Anti-AdoHcy antibodies were used as the recognition agent. The fluorescein-AdoHcy conjugate served as a label. This compound competed with AdoHcy, a product of methyltransferase activity, for binding to the enzyme. As a result of the competitive interaction, the concentration of the label increases and, consequently, the FP decreases. The analysis results showed that the antibody is more than 150 times preferable for binding AdoHcy than AdoMet. Mestas et al. [108] proposed a simple, high-throughput assay based on FP to determine the elongation activity of nucleic acid polymerases. The analysis is based on the intermolecular interaction between the 5′-labeled chain of the template and the fluorescent dye, which causes a change in the conformation of the nucleic acid. If the oligonucleotide is short enough, the FP can also be used to detect binding prior to elongation activity. The polymerase assay allows to screen compounds that inhibit nucleic acid binding or polymerase extension activity.

## 10. Towards Multiplex Analysis

Assays of several compounds having similar or different chemical natures are often necessary. Therefore, a natural tendency has been to create multiparametric systems that actively attract the attention of researchers through commercially profitable implementation that reduce reagents, the total cost of analysis, and the detection time. One advantage of the FPIA is the ease of implementation of multiparameter analysis. Theoretically, to implement this approach to increase the number of analyzed compounds, it is sufficient to determine an additional analyte in an adjacent well of a microplate, or add another fluorophore, or use group-specific receptors. Despite the theoretical simplicity of the multiplex PFIA, in practice, single works have been implemented. Boroduleva et al. [109] developed FPIA for two pesticides, triazophos and carbaryl, in wheat grains using the Ellie Sentry 200 handheld FPIA instrument. Using ethylenediamine-fluorescein-thiocarbamyl as a dye made it possible to increase the sensitivity of the analysis with a minimum consumption of reagents. The authors have developed a fast and high-throughput sample preparation procedure for the parallel determination of the both analytes in aliquots of the same sample. LoDs of triazophos and carbaryl were 40 and 20 μg/kg, respectively.

Zhang et al. [110] described the implementation of multiplex FPIA for simultaneously detecting three pesticides (triazophos, parathion, and chlorpyrifos) in various agricultural products. The authors use fluorescently labeled oligonucleotides as an indicator and use AuNPs to increase the assay sensitivity. Simultaneously, the reaction was carried out with specific antibodies and labeled nucleotides immobilized on the surface of nanoparticles. Three dyes (6-FAM, Cy3, and Texas red) with high fluorescence intensities and a small excitation / emission crossover of wavelengths were used. For these three analytes, LoDs were 0.007, 0.009, and 0.087 μg/L, respectively. Guan et al. [57] proposed competitive FPIA for simultaneous monitoring of BPA and its analogues using Dex-FL as a dye. Under optimized conditions, four bisphenols were detected at concentrations meeting European Union regulations. FPIA showed LoDs of 0.08–0.49 mg/L for different bisphenols. With high sensitivity, the developed assay has demonstrated good potential for rapid screening of bisphenols in food.

The aim of another study [56] was the simultaneous detection of BPA and its analogs. The developed FPIA demonstrated LoDs of 0.21–1.03 μg/g for four compounds, which may meet the requirements of environmental monitoring. In this study, a broad specificity receptor protein for bisphenols was obtained that was used for FPIA in soil samples. Li et al. [111] described a homologous and high-throughput multi-wavelength FPIA for the multiplex detection of three mycotoxins of the Fusarium genus: deoxynivalenol (DON), T-2 toxin, and fumonisin B1 (FB1). Three fluorescent dyes were used as indicators. The role of the antigen-binding component was played by specific antibodies. Under optimal conditions, LoDs were 242.0 μg/kg for DON, 17.8 μg/kg for T-2 toxin, and 331.5 μg/kg for FB1. The total analysis time was less than 30 min.

The enantioselective assays and sensors have attracted a lot of attention for the detection of enantiomeric impurities. Chovelon et al. [45] demonstrated the previously described strategy for the analysis of the so-called aptamer kissing complex (AKC) and its possible implementation in simultaneous enantiomer quantification and enantiopurity analysis. D- and L-AVP were used as model enantiomeric targets. Engineered D- and L-AVP aptamers (aptaswitches) were used as recognition units, while fluorescein-labeled or Texas Red D- and L-hairpins (aptakiss) served as probes for enantiomer-dependent AKC formation. Orthogonal fluorescence anisotropy signal transmission at two emission wavelengths allowed simultaneous determination of AVP enantiomers in one sample in a high-performance microplate format. The enantioselective AKC sensor has also been shown to detect enantiomeric impurities as low as 0.01%.

## 11. Switched FP and its Analytical Use

**Figure 9:**
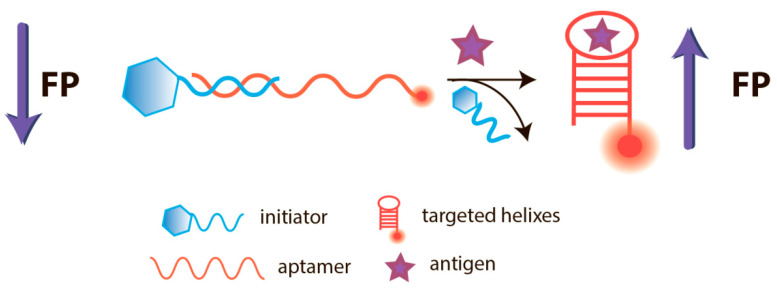
Principle of the switched FP

FP and FA are commonly used as two interchangeable methods. Combining the advantages of aptamers as receptor agents and FA, this method allows the detection of small molecules with higher sensitivity and removes some of the limitations inherent in traditional immune assay formats. Aptamer structure switching FA assays for detecting low molecular weight compounds are based on changes in anisotropy caused by competition between aptamer-target binding and a conformationally altered aptamer-complementary DNA (cDNA) complex [112]. A significant increase in the FP change is due to the large difference in the rates of rotation of the free aptamer and the aptamer-cDNA complex (Figure 9).

The principle of switching the structure of aptamers is that exonuclease I (Exo I) destroys ssDNA in the direction from 3′ to 5′ and does not affect dsDNA. Exo I plays the main role in this reaction, which makes it a versatile drug for the detection of compounds of different sizes and natures. The hybridization chain reaction (HCR) proceeds without the participation of enzymes. The initiator starts a cascade of hybridizations of two freely used DNA hairpins, resulting in the formation of long cuts. The molecular weight of the HCR products and the concentration of the initiator are directly related. Its ability to immobilize indicator molecules on hairpins makes HCR a promising method for signal amplification in analytical applications. So far, targeted reworking of the Exo I and HCR has become widespread. The paper does not provide experimental confirmation of the simultaneous use of aptamers and double amplification. Isothermal amplification techniques have proven to be powerful and promising tools that perform amplification with high efficiency and high speed at a constant temperature without thermal cycling.

Zhao et al. [27] reviewed the most recent advances in affinity determination using aptamers and FP. Much of the interest is due to intermolecular interactions and binding identification. Using FP, you can efficiently register the binding of aptamers, DNA with target analytes. Aptamers offer several advantages, screening for aptasensor with signal amplification is based on the above-described reaction catalyzed by exonuclease I. The authors have proposed to use this principle to determine the antibiotic chloramphenicol. After the HCR initiator blocks the aptamer-binding domain, the aptamer changes its tertiary structure in the presence of chloramphenicol, which leads to DNA dissociation. In the next step, the released aptamer is recognized and cleaved by Exo I to release chloramphenicol. Under optimal conditions, the aptasensor shows a linear range from 0.001 to 100 nM chloramphenicol and a LoD of 0.3 pM.

Perrier et al. [113] proposed new FP-based aptamer assays for low molecular weight antigens. The signal-enhancer oligonucleotide (SEO) served as an analyte detector. By targeting specific regions of the signaling DNAs, the binding of SEO to free aptamer triggers disruptions in both the internal versatility of the DNA and the localized environs of the dye when the free analyte enters the duplex structure. This effect determines the increased FP variation between duplex and target bound states of the aptamer. The authors used fluorescein and Texas Red dyes to record pre-structured (adenosine) and unstructured (tyrosinamide) aptamers. Li and Zhao [114] proposed an FP assay with structure-switching aptamer specific to mycotoxin AFB1. They used a TMR-labeled aptamer and its cDNA with tandem extension of G bases as a detection label. This approach allows achieving high sensitivity and selectivity in the detection of AFB1. Co-hybridization of aptamer and cDNA results in TMR approaching guanine (G) repeating bases and induces high FP value due to TMR-G interaction and limited rotation of TMR. This analysis revealed AFB1 with a LoD of 125 pM and a dynamic range of 125 pM to 31.2 nM.

Taking advantage of the aptamers, Liu and Zhao [43] developed a simple and fast FP analysis for BPA. The 35-mer DNA aptamer against BPA plays a major recognition role. If BPA is present in the sample, the conformation of the TMR-labeled aptamer changes due to the interaction of the guanine base. This reaction leads to a change in the fuel assembly signals. The LoD of BPA was 0.5 μmol/L under optimized conditions. Li et al. [64] offered fast and sensitive OTA based on FP detection and aptamer switching. The authors synthesized the OTA conjugate with rhodamine B, which was used as a competitor in the FP-based aptamer assay. The role of a receptor agent was played by an OTA-specific aptamer capable of participating in the signal switching on and off. For further improvement of the assay sensitivity, the authors used the SA molecule to increase the molecular weight and decrease the rotation rate of the dye-labeled agent. This approach provided a LoD of 2.5 nM and a more noticeable decrease in FP. In addition, the fluorescent probe can interact with Tween 20, which causes a higher FP than the aptamer-fluorescent probe complex. LoD of OTA was 10 nM.

Li and Zhao [115] also used a two-step FP sensitization because they were simultaneously using a structure-switching aptamer and SA molecules. This approach is based on the proximity effect to reduce the rotation of the fluorophore. The formation of a complex of the aptamer with the target low molecular weight causes the displacement of the cDNA labeled with SA, which leads to a significant decrease in FP. The proximity of SA to FAM in a duplex allows one to obtain significant FP changes when determining low molecular weight compounds. This method allowed the detection of 60 pM AFB1, 1 nM of OTA, and 0.5 μM of ATP. Goux et al. [116] described the use of peptide nucleic acids (PNAs) served as an alternative to DNA probes in fluorescence polarization tests of switchable aptamers. They first investigated the effect of PNA chain length, the nature of the dye, and buffer conditions on assay efficiency. Two methods were used in the work. The first is based on PNA / aptamer hybridization. The second is based on the formation of an analyte-marker complex. The linear range for ATP is 1 to 25 μM, with a LoD of 3 μM, five times lower than competing models.

## 12. FPA with Signal Enhancement

Despite the successful implementation and widespread use of the aptamer-based FP assay, this method does not always satisfy the set objectives of sensitivity. When using most of the previously considered assay schemes, the principle is to bind recognition molecules to large mass species, which makes it possible to increase the FP difference between probes without a ligand and probes with a ligand. To this end, various nanomaterials and nucleic acid binding proteins can be integrated. We’ll discuss these different strategies below.

In recent years, numerous approaches have been proposed to reduce the LoD. However, it is important that achieving this goal does not involve significant complication of the analysis and its integration with complex and expensive instrumentation. When quantitative results are required, portable optical detectors, cameras, and smartphones are used to determine the intensity of the optical signal. Using these detectors does not significantly complicate the analysis. As a promising approach, FP is a versatile and excellent tool for assessing the speed of molecules of molecules of different natures in a homogeneous environment [8]. In contrast to the fluorescence intensity, FP is practically independent of the concentration or amount of fluorophores, but strongly depends on the size or molecular weight of the molecules with the fluorophore. Last 10 years, significant amount of work has been made in the field of FP with the introduction of some nanomaterials as FP amplifiers, which thus significantly improves the detection sensitivity, and FPs based on nanomaterials are currently successfully used in immunoassay of proteins, nucleic acids, small molecules, and metal ions. Nanomaterial FP provides a new kind of strategy for developing fluorescent sensors. The methods are based on either a covalent/bioaffinity complex or adsorption immobilization of the receptor molecules on a nanomaterial [117].

### 12.1. Nanoparticles as Signal Enhancers

Currently, nanomaterials are widely used for analytical purposes [118]. For the specific purpose of FP analysis, the main apparent advantage of nanomaterials lies in their high volume characteristics. In addition, nanoparticles, having a well-developed surface, easily absorb not only large proteins but also small nucleic acids, such as functional nucleic acids (FNAs) [119]. Two areas of application of nanoparticles in an FP system are worth noting for amplifying an analytical signal. First, nanomaterials can be used directly to increase the mass of the complex and, therefore, reduce the rotation rate. The second role of nanoparticles is to transfer signaling markers through adsorption immobilization on their own surface. This section proposes to consider options for using nanoparticles of various natures in FP analytical systems, such as AuNPs, QDs, carbon particles, magnetic nanoparticles (MNPs), and some other nanomaterials. The unique FP amplifiers are QDs characterized by long fluorescence lifetime, relatively small size, but high mass.

Thus, a complex formed by a protein-QDs and a digoxin-ATP aptamer initially binds to a specific antibody. Analyte-induced displacement of the FP causes the receptor to be removed and then reduced in mass [3]. Samokhvalov et al. [120] proposed AuNPs as carriers to reduce the LoD of the FP-based aptamer assay. This work demonstrates the advantages of AuNPs as unified, stable, and simply modified carriers of aptamers. The assay was performed using AuNPs with an average diameter of 8.7 nm and OTA as the target analyte. Finally, the test was checked for the OTA control in the flavored white wine. The achieved LoD was 2.3 μg/kg, which is 25 times lower than the native aptamer. The assay duration was 15 min. Ye et al. [121] proposed two options for increasing the sensitivity of the FP-based aptamer assay. The broad-spectrum ssDNA aptamer, capable of binding to four different lipopolysaccharide (LPS) sources, was truncated. The data obtained indicate that the aptamer of 27 nucleotides retains the ability to a wide class of compounds and has a higher affinity. An FP-based graphene oxide aptamer assay using a FAM-labeled aptamer can detect LPS from *Salmonella entericaserotype typhimurium*, *Pseudomonas aeruginosa* 10 and *Escherichia coli* 055:B5 with improved performance. The analysis can be done within 30 min; the LoDs were 38.7, 88.0 and 154 ng/mL, respectively.

Huang et al. [122] developed two FP-enhanced aptasensors. The proposed sensors are based on signal amplification by an enzymatic click and nanodispersed graphene oxide (GO), which make it possible to record biomolecules in a solution. The first approach involves the binding of the aptamer to the target analyte through stable complexation between the aptamer receptor and the DNA-fluorophore complex. The latter, in turn, is adsorptively bound to GO and actively participates in the formation of the duplex region of DNA containing the capture site through the enhancement of base stacking. In the second method, the target analyte provokes the assembly of two subunits of the aptamer into the aptamer-analyte complex, followed by hybridization with the participation of the DNA-fluorophore complex. The latter, in turn, is adsorptively bound to GO and actively participates in the formation of a duplex DNA region containing a capture site. The formation of a duplex region of DNA triggers an enzymatic click process, which results in the release of short DNA fragments with the dye from GO, causing a significant FP decrease. Using this approach, the assay sensitivity can be improved by four orders of magnitude.

Chen et al. [123] developed a FP aptasensor based on a conjugate of MNP with polydopamine for detection of recombinant human erythropoietin alpha (rHuEPO-α). The enhanced FP signal is due to high masses of protein and MNP–PDA. This analysis can be used to separate or reuse targets based on the magnetic properties of MNP–PDA. The LoD of rHuEPO-α was 0.12 pM, which is four orders of magnitude lower than in the original analysis. 

Despite the advantages of nanoparticles, their use in increasing the sensitivity of FP analysis has limitations. The disadvantages of nanoparticles include uncontrolled size (spread in mass). They are also capable of quenching fluorescence to a large extent, which seriously reduces the accuracy and sensitivity of the FP method. Nanomaterials realize cascade amplification due to their joint application with proteins.

### 12.2. Proteins-Based Signal Amplification

The advantages of FPIA are that it is performed in one step with fast diffusion-independent interactions and that the analytical signal can be recorded directly during complex formation. Its simplicity and speed have made this method popular for solving various research and applied problems. However, the vast majority of FPIAs are based on the use of antibodies [5]. For aptamers, the increase in mass resulting from the formation of the analyte-receptor complex is much smaller, which reduces the assay’s sensitivity. To increase the sensitivity of the FP aptamer assay, the incorporation of aptamers into complexes with proteins, the so-called anchor modules, was recently proposed. The use of complexes of a biotinylated aptamer with SA and a SA-IgG conjugate showed that this approach significantly reduces the LoD. Until recently, an FP analysis based on the incorporation of aptamers into protein complexes was implemented, using the ATP-binding aptamer as an example. Anchor modules have also been used in the FP test for pyrethroid insecticides using nanobodies. However, protein conjugates cannot be considered optimal because of the variability of their stoichiometry and the risk of aggregation. The simplest version of FP assay enhancement is based on the ability of the SSB to bind the fluorescently labeled aptamer only in its free, unstructured state. The presence of the target analyte and its interaction with the aptamer leads to the release of the receptor from the protein surface because of the structuring of the FNAs. This interaction significantly decreases the FP level because of a decrease in mass (~70 kDa) as well as a possible increase in the segmental movement of the indicator in the form of ligand. Minimal quenching and scattering effects compared to non-covalent nanomaterials increase analytical accuracy [117].

A similar model can be realized using a biotin–SA bridge (~60 kDa) or a SA-IgG complex (~200 kDa). Instead of complementary strand, a fluorescent analog can be used [42]. More complex is the signal transduction mechanism for thrombin detection. This system contains a fluorophore-aptamer complex consisting of two linked receptor units, one of which is specific to a low molecular weight antigen, and the other targeted to the thrombin. A strand partially complementary to the two single sequences denatures the entire DNA structure. In the presence of an analyte, the conformational rearrangement of the low-molecular-weight aptamer causes the dissociation of the complex which is free for further complexation with the protein. This results in a dual binding mechanism, the first of which reveals the target’s presence and the second of which potentially improves the change in probe mass. However, only a small increase in FP is achieved because of the protein’s relatively limited size and because of some effects of local movement of the fluorophore. Finally, the FA response can be enhanced by introducing SA-based mass amplification into the assay [46]. In general, compared with the amplification of the nanomaterial, the test response and LoD (nM in the best cases) improve to a lesser extent. Another limitation on the use of protein enhancers in FP analysis is their lot-to-lot and vendor-to-vendor variability, as well as the need to work under strongly fixed conditions consistent with the properties and stability of biomolecules.

Li and Zhao [115] have described an analysis with switching aptamer structure for low weight molecules using SA as signal amplifier. The SA molecule promotes the formation of close contact to decrease the rate of the fluorophore rotation. In this construct, cDNA covalently linked to SA hybridizes to a FAM-labeled aptamer, bringing FAM closer to SA and providing a much higher FP value because of limited rotation of FAM. Binding of the complex with a low molecular weight antigen and aptamer causes a shift in the SA-labeled cDNA and a significant decrease in FР. The main phenomenon is the nearness of the SA to the FAM in duplex, which allows large changes in FA in target acquisition. The method enables detection of 60 pM of AFB1, 1 nM of OTA, and 0.5 μM of ATP. Ma et al. [124] described the FP aptasensor based on PCR and SA as dual FP amplifiers for detecting chloramphenicol leftover in food. The label-free aptamer interacted with the analyte and the resulting complex was used as a template for PCR. Then a forward primer labeled with FAM and a reverse primer labeled with biotin were added to amplify the template and primer labeled with FAM. The molecular weight of the FAM-labeled primer and the corresponding FP increased rapidly. After the addition of SA to the mixture, the biotin-containing preparations formed a large complex in size and mass, resulting in a much higher molecular weight. Under optimal conditions, a wide linear detection range of 0.001–200 nM was achieved.

Li et al. [47] proposed increasing the size and mass of the analyzed complex for FP-based detection of microRNA. The proposed method can be used for the detection of targets in tumor cell lysates without any tedious pretreatment and with linearity in the range from 10 pM to 0.5 nM with LoD up to 3.4 pM. Liu et al. [125] proposed the use of a two-dimensional DNA nanosheet (DNS) for the amplification of FP. This DNA structure causes fluorescence quenching. A fluorophore-labeled receptor DNA is immobilized on the DNS surface by hybridization with a DNA handle (hDNA). This method of bond formation contributes to a significant increase of FP. After the addition of the analyte, the receptor DNA, because of the high affinity between hDNA and the analyte, is released from the surface of the DNS, thus decreasing the FР. The determined compound was registered with a significantly reduced FA value. The reached linear range was 10–50 nM and LoD was 8 nM for single-stranded DNA detection.

Separately, the increase in the sensitivity of FPIA from the use of enzymes is worth noting. The use of catalytic recirculation systems based on enzymatic cascades was proposed. These reaction schemes have shown their effectiveness in complex multiparameter systems. The latest generation of PFIA amplification methods is based precisely on the combined effect of weight gain and enzyme-based analyte recirculation. The approaches developed based on this principle can be classified according to the enzymatic reactions involved. Chen et al. [126] proposed the assay in which in the presence of small target molecules, DNA 1 labeled with FAM and DNA 2 labeled with biotin were ligated to obtain integrated DNA. This method can detect 0.05 to 1 μM ATP with 41 nM LoD and detect 0.01 to 1 μM NAD^+^ with 6.7 nM LoD.

## 13. Overall Estimation and Validation of FP-Based Assays

Validation is key issue in the quality assurance system for quantitative analyses confirming acceptance of the proposed assay to the requirements for its specific application. The obtained results indicate perspective of new assay technique and its place among alternate ones. In this connection, common properties of the assays based of the use of FP as the registered signal should be characterized as well as the data about their detailed validation. Since most of the new analysis formats discussed above are at the stage of laboratory development of prototype analytical systems, most of the validation work belongs to the FPIA variants. However, they demonstrate a number of properties common to all methods considered in the review.

A specific feature of FP and FA is that they are dimensionless parameters reflecting the ratio of the concentrations of reagents in the reaction medium. Due to this, the results of the analysis are characterized by high reproducibility and do not depend on the peculiarities of testing in different laboratories (temperature conditions, impurities in the reaction medium, etc.). The rapid course of reactions in solution in the absence of diffusion hindrances makes it possible to detect the assay results in an equilibrium mode and thereby exclude the influence of fluctuations in the recording time on the reproducibility and accuracy of measurements.

As a result, the accuracy of measurements is primarily determined by the accuracy of reagents’ dosing and depends on the total volume of the reaction mixture. When analyzing in cuvettes for FPIA devices from Abbott or other manufacturers (the volume of the reaction mixture is 0.5–1 mL), the relative standard deviation (RSD) usually varies in the range of 5–7%. In the case of microplate photometers with the function of fluorescence polarization registration (the volume of the reaction mixture in a microplate well is 0.1–0.2 mL) the RSD can reach 10%,

The accuracy of determining the analyte concentration also depends on the calibration concentration dependence, which should ensure both low LoD and a large range between the minimum and maximum recorded values of polarisation. Tus, the assay conditions (concentrations of reactants, composition of reaction medium, etc.) should be optimized. The main criterion for evaluating the effectiveness of the proposed assay protocol is Z’ factor, a measure of statistical effect size, which is calculated by the Equation (5) [127]:

(5) $$Z^{\prime }=1-\frac{3\left(\sigma_{p}+\sigma_{n}\right)}{\left(\mu_{p}+\mu_{n}\right)}$$

where σ*_p_* and σ*_n_* are the standard deviation of positive and negative controls, respectively, and μ*_p_* and μ*_n_* are the FP values of positive and negative controls, respectively.

The value of Z > 0.5 (its theoretical upper limit is equal to 1) indicates successful optimization. For example, Liu et al. [127] varied not only the concentrations of components, but also the distance between the working solution and the reading element. This approach allowed to reach Z’ 0.7–0.8.

The used receptor molecules (antibodies, aptamers, etc) are responsible for specificity of FPAs. In contrast with confirmatory analytical techniques (chromatography, electrophoresis etc.) the receptor-based assays including FPAs considered here generate one integrated signal for structurally similar compounds that can bind with the chosen receptor, so the lists of cross-reacting compounds are identical for various assay formats based on the same receptors, and values of cross-reactivity are close.

An often statement is that FPA is inferior in sensitivity to such methods as ELISA [121]. It is a result of direct registration of the formation of immune complexes without enhancing processes. However, for high molecular weight compounds, such as antibodies, FPA sensitivity has been shown, comparable to ELISA [122]. The estimation of FPA properties given below is summarized in Table 2.

Depending on the tested analytes, FPAs are the most commonly compared with ELISA as the most widespread immunoanalytical technique and with different chromatographic techniques as typical confirmatory techniques. The general opinion is that immunoassays should provide better correlation due to the use of the same receptor molecules. In fact, R^2^ for immunoassays may also differ greatly, taking into account different principles of the formation of detectable complexes and the use of antibody preparations with different affinities and specificities. Some data about the correlation with other analytical techniques are collected at Table 3.

## 14. Equipment for FPA

For a long time, the need for special equipment limited wide acceptance of FPIA. Abbott was one of the first to create a fully automated TDx analyzer and for a long time remained a monopolist in the market for FPIA instrumentation and reagent kits. Currently, there are many more devices for FPIA: Sentry (Ellie LLC, Germantown, WI, USA), Beacon 2000 (PanVera, Madison, WI, USA), Tecan Safire 2 (Tecan, Männedorf, Switzerland), Victor (LKB-Pharmacia, Brentwood, TN, USA), Wallac Victor2V (Perkin Elmer, Beaconsfield, UK), and others. In addition, portable FP detectors manufactured by Ellie LLC (Germantown, WI, USA) and BMG Labtech (Ortenberg, Germany) have appeared.

Thus, Ellie LLC produces portable detectors for FPIA and compatible reagent kits for animal health diagnostics, including tests for brucellosis, tuberculosis, progesterone in milk. An intereasting feature of their kits is the use of fluorescein-labeled antigenic peptides to detect protein antigens by competitive scheme of FPIA. In their studies, Boroduleva et al. used an Ellie Sentry 200 portable device, for implementing FP assays of different contaminants [109,135,136]. Three derivatives of fluorescein were compared to ensure the best assay sensitivity and minimum reagent consumption in the FP-based determination of thiabendazole and tetraconazole fungicides [135]. The best analytical parameters were achieved using 4-aminomethylfluorescein as a tracer. The LoDs of thiabendazole and tetraconazole in wheat were 20 and 200 g/kg, respectively. The same equipment was used for the determination of pesticides carbaryl and triazophos in wheat grains [109]. Tracer labeled with fluorescein derivative—ethylenediamine thiocarbamyl—was applied in a FPIA. The LoDs for triazophos and carbaryl were 40 and 20 mg/kg, respectively. A Sentry 200 portable device was used in the FPIA procedure for the determination of 2,4-dichlorophenoxyacetic acid in cereal grains. 4-Aminomethylfluorescein-based tracer ensures the highest sensitivity of the analysis, namely, 40 ng/g with the analytical range of 80–1000 ng/g [136].

New approaches for detecting FPIA results are described in several publications. Smart phones can be used as a part of sensor devices after appropriate add-ons. Thus, Zhao et al. [137] developed a smartphone-based sensor based on the camera of a smartphone and a 3D-printed compact holder. The sensor chip in the smartphone camera has two regions that can detect parallel and perpendicular polarized emissions. A special software app can assess the average intensity and the degree of polarization. A competitive FPIA of prostaglandin E2 demonstrated the LoD of 1.57 ng/mL. Wargocki et al. presents a simple cell phone-based portable bioassay platform, which can be used with fluorescent assays of collagenase and trypsin using fluorescein [138]. The system includes a box for the readout in the dark, a smart phone camera, a tablet, and a polarizer. The tablet screen acts as an excitation source. The assay sensitivity was comparable to that obtained by a microplate reader: namely, the lowest measured content in the sample was 0.938 μg and 930 pg for collagenase and trypsin, respectively. The developed sensor system was sensitive enough for the point-of-care medical diagnostics of clinically relevant conditions.

Wakao et al. [139] constructed an inexpensive portable FP analyzer with a microdevice for high-throughput FP immunoassays for mycotoxin DON. The analyzer was able to simultaneously detect 96 samples requiring only 1 nL as a sample volume. The authors demonstrated that the developed device was suitable for on-site measurement and point-of-care testing. Further, the authors reported the successful development of a rapid detection of anti-H5 subtype avian influenza virus antibody in serum by FP assay using the same portable analyzer [140]. The analysis was carried out within 20 min, and only 2 μL of the sample was required to fulfill it. Choi et al. [141] proposed a droplet-based microfluidic chip for the analysis of deoxyribonuclease (DNase) activity that involved no conjugation with dyes. Ethidium bromide was used as a DNA intercalating reagent and a fluorescent reporter; therefore, no prior conjugation or modification of DNA was required. The developed test system allowed for the determination of half-maximal inhibitory concentration of ethylenediaminetetraacetic acid for the inhibition of DNase 1 activity to be 1.56 ± 0.91 mM. Schrell et al. [142] developed a real-time system for insulin monitoring based on a microfluidic online FA immunoassay. It has a LoD of 4 nM and is suitable for use by non-specialized laboratories.

The price of instruments for measuring the polarization of fluorescence is steadily decreasing. Many devices, such as Spectromax M5 (Molecular Devices, San Jose, CA, USA), PolarStar Optima (BMG LabTechnologies, Ortenberg, Germany), Chameleon (Hidex, Turku, Finland), and Zenyth (Anthos Labtec Instruments, Wals, Austria), detect FPIA results in 96-well ELISA plates, where FP is measured after excitation/emission with a vertical beam. For this FPIA format, black or white plates are used to exclude interference from adjacent wells. Such plates, like conventional tubes, can be reused to produce FPIA. Recently, 384-well plates have also been used for FPIA, which significantly reduces the consumption of reagents (only a few μL is required) and increases the productivity of determinations. The usage of a Zenyth 3100 microplate reader was described in a series of papers [11,143,144]. Thus, Zvereva et al. used this equipment for FPIA of an alkaloid colchicine (COL) and ractomanine (RAC), respectively. The combination of the polyclonal antibody and the antigen labeled with FITC allowed detection COL with a LoD of 1.8 ng/mL within 10 min [143]. For RAC, the optimal assay conditions enabled detection of 1 ng/mL also within 10 min [144].

## 15. Prospects of FPIA

New investigations demonstrate ways to overcome commonly stated limitations of traditional FP-based assays. Various cascade enhancing formats provide extremely sensitive detection of analytes being comparable with other immunotechniques. The influence of matrix compounds on the assay results may be eliminated by special treatment procedures developed for various tested substances or by choice from the extended row of available fluorophores. To extend the applicability of FPIA from low molecular weight compounds to proteins, labeling of antigenic peptides has been successfully used.

Recent developments presented in this review indicate a significant variety of analysis formats in which the registration of fluorescence polarization can be used effectively. The development of new protein and oligonucleotide receptor molecules by their molecular designs makes it possible to further expand the fluorescence polarization-based assays’ application to a wide range of detectable compounds to combine the rapidity and simplicity of polarization fluorescence detection with improvements in the assays’ sensitivity and selectivity. Assays based on the registration of fluorescence polarization can be implemented using different fluorophores, and their wide screening with the selection of the most effective molecules is becoming a task that is much in demand. Schemes for amplifying the analytical signal using additional carriers and chains of sequential interactions are being developed successfully, which will also make it possible to achieve lower detection limits and increase the competitive potential of the fluorescence polarization-based assays. An important factor determining opportunities for further application of these methods is the development of portable devices for the registration and processing of optical signals, including those based on mass-produced communication devices.

## Figures and Tables

**Table 1:** Aptamer-based FPAs considered in this review.

Analyte/Medium	Aptamer Used	Limit of Detection	References
Bisphenol A/tap water	5′-CCG CCG TTG GTG TGG TGG GCC TAG GGC CGG CGG-(TMR)-3′	0.5 µM	[44]
Cocaine/serum and urine	5′-GAC AAG GAA AAT CCT TCA ATG AAG T(TMR)GG GTC-3′	5 µM	[45]
D- and L-arginine vasopressin / serum	5′-TCA CGT GCA TGA TAA CAC TCC CAT TCT GAG TTG CTG TGT GCC GAT GCA CGT GA-fluorescein(or Texas-Red)-3′	40 nM; 20 nM	[46]
Adenosine/cell culture medium	5′-fluorescein-AGdT GAA CCT GGG GGA GTA TTG CGG AGG AAG GT-biotin-3′	500 nM	[47]
MicroRNA/tumor cell lysates	5′-GGT TGG TGT GGT TGG TCA ACA TCA GTC TGA TAA GCTA-fluorescein-3′	3.4 pM	[48]
Ochratoxin A/buffer	5′-fluorescein-GGG AGC ATC GGA CTT TTT TT-biotin-3′	25.2 fM	[49]
Ochratoxin A/white wine	5′-GAT CGG GTG TGG GTG GCG TAA AGG GAG CAT CGG ACA-biotin-3′	2.8 nM	[11]
Adenosine/cell culture medium	5′-GGT TGG TGT GGT TGG ACC TGG GGG AGT ATT GCG GAG GAA GGT-fluorescein-3′	0.5 μM	[43]
Cocaine/urine	5′-fluorescein-AGAC AAG GAA AAT CCT TCA ATG AAG TGG GTC G GGT TGG TGT GGT TGG-3′	0.8 μM	[43]
Adenosine/serum	5’-TTG TTA CCT GGG TTT TTT TTTT-SH-3’	20 pM	[50]

**Table 2:** Estimation of FPA parameters (using data from [128,129,130]).

Parameter	Comparative Characterization
Rapidity	High (5–10 min for typical protocols)
Labor intensity	Low (one-step assays with minimal manipulations)
LoD	Comparable with other immunotechniques (individual for analyte)
Working range	Variable (for competitive assay—about an order of magnitude)
Reproducibility	High (due to dimensionless registered FP and FA values)
Accuracy	Depends on the reaction volume

**Table 3:** Correlation of the FPA and other methods.

Analyte	Reference Method	R^2^	Reference
**o** **ther immune techniques**			
N-terminal kinase 3	ELISA	0.9555	[131]
p38a mitogen-activated protein kinase	ELISA	0.8447	[131]
Brucellosis	ELISA (two formats)	0.8564/0.9116	[132]
Gentamicin	immunochromatography	0.976	[133]
**o** **ther non- immune techniques**			
Bisphenol A	HPLC	0.9636	[56]
Fluoroquinolones	HPLC	0.9665	[132]
T-2 and HT-2 toxins	UHPLC	0.953–0.998 depending on matrix	[134]
Triazophos	Gas chromatography	0.9680	[128]

## Abbreviations

Adenosylhomocysteine (AdoHCys); adenosylmethionine (AdoMet); adenosine diphosphate (ADP); aflatoxin B1 (AFB1); aptamer kissing complex (AKC); adenosine monophosphate (AMP); adenosine triphosphate (ATP); gold nanoparticles (AuNPs); arginine vasopressin (AVP); bisphenol A (BPA); complementary DNA (cDNA); carboxymethylcellulose (CMC); C-reactive protein (CRP); dideoxyribonucleoside triphosphate (ddNTP); dexamethasone fluorescein (Dex-FL); DNA nanosheet (DNS); deoxynivalenol (DON); double-stranded DNA (dsDNA); enzyme-linked immunosorbent assay (ELISA); fluorescence anisotropy (FA); carboxyfluorescein (FAM); fluorescein isothiocyanate (FITC); fluorescence polarization (FP); fluorescence polarization assay (FPA); fluorescence polarization immunoassay (FPIA); fumonisin B1 (FB1); functional nucleic acids (FNAs); graphene oxide (GO); hemagglutinin (HA); hybridization chain reaction (HCR); immunoglobulin G (IgG); Michaelis constant (KM); limit of detection (LoD); lipopolysaccharide (LPS); molecularly imprinted polymers (MIPs); magnetic nanoparticles (MNPs); Nile blue (NB); nitrobenzoxadiazole (NBD); ochratoxin A (OTA); Quenchbody (Q-body); quantum dots (QDs); phthalate esters (PAEs); polymerase chain reaction (PCR); polydopamine (PDA); peptide nucleic acids (PNAs); relative standard deviation (RSD); streptavidin (SA); Systematic Evolution of Ligands by Exponential Enrichment (SELEX); signal-enhancer oligonucleotide (SEO); single-stranded DNA binding protein (SSB); tetramethyl rhodamine (TMR).
